# Predicting spinal profile using 3D non-contact surface scanning: Changes in surface topography as a predictor of internal spinal alignment

**DOI:** 10.1371/journal.pone.0222453

**Published:** 2019-09-26

**Authors:** J. Paige Little, Lionel Rayward, Mark J. Pearcy, Maree T. Izatt, Daniel Green, Robert D. Labrom, Geoffrey N. Askin

**Affiliations:** 1 Biomechanics and Spine Research Group, Institute of Health and Biomedical Innovation, Queensland University of Technology, Brisbane, Australia; 2 Sealy of Australia, Wacol, Australia; 3 Wesley Hospital, Brisbane, Australia; 4 Mater Health Services, Brisbane, Australia; University of California Davis, UNITED STATES

## Abstract

**Introduction:**

3D non-contact surface scanners capture highly accurate, calibrated images of surface topography for 3D structures. This study sought to establish the efficacy and accuracy of using 3D surface scanning to characterise spinal curvature and sagittal plane contour.

**Methods:**

10 healthy female adults with a mean age of 25 years, (standard deviation: 3.6 years) underwent both MRI and 3D surface scanning (3DSS) (Artec Eva, Artec Group Inc., Luxembourg) while lying in the lateral decubitus position on a rigid substrate. Prior to 3DSS, anatomical landmarks on the spinous processes of each participant were demarcated using stickers attached to the skin surface. Following 3DSS, oil capsules (fiducial markers) were overlaid on the stickers and the subject underwent MRI. MRI stacks were processed to measure the thoracolumbar spinous process locations, providing an anatomical reference. 3D coordinates for the markers (surface stickers and MRI oil capsules) and for the spinous processes mapped the spinal column profiles and were compared to assess the quality of fit between the 3DSS and MRI marker positions.

**Results:**

The RMSE for the polynomials fit to the spinous process, fiducial and surface marker profiles ranged from 0.17–1.15mm for all subjects. The MRI fiducial marker location was well aligned with the spinous process profile in the thoracic and upper lumbar spine for nine of the subjects. Over the 10 subjects, the mean RMSE between the MRI and 3D scan sagittal profiles for all surface markers was 9.8mm (SD 4.2mm). Curvature was well matched for seven of the subjects, with two showing differing curvatures across the lumbar spine due to inconsistent subject positioning.

**Conclusion:**

Comparison of the observed trends for vertebral position measured from MRI and 3DSS, suggested the surface markers may provide a useful method for measuring internal changes in sagittal curvature or skeletal changes.

## Nomenclature

For the purpose of this study, the term **profile** refers to quantitative marker locations and **curvature** or **contour** refers to spinal shape.

## Introduction

Non- contact surface scanners capture 3D images of surface topography and shape. In doing so, they enable the virtual analysis of real objects to be conducted in order to gather qualitative and quantitative data on shape, size and colour of the object. From this, virtual reconstructions of a 3D object can be created with a high level of accuracy (in the order of a micron, depending on the scanner) to generate a dimensionally accurate, calibrated reconstruction. Such calibrated reconstructions provide a dimensionally accurate record of the object of interest at a particular point in time.

3D non-contact scanning using optical or light-based scanners has been utilised since the 1980s [1]. The technology has seen broad application in fields including reverse engineering and design of machinery parts, spatial reconstructions to capture accurate 3D representation of crime scenes for forensic investigations [2], computer graphics applications for gaming and movie production (www.artec3d.com/applications), and artistic or historically-driven reconstructions of physical artefacts (eg. paleantology) [3]. In terms of clinical applications, much interest surrounds the use of non-contact scanners to capture physical surface anatomy, whether this be for application in creating orthotic devices [4], in creating bespoke reconstructions for plastic surgery or in measuring surface contours [5] for anatomy such as the torso and spine.

In recent years, there’s been an interest in using 3D scanning to measure spinal shape for biomechanical or ergonomic assessments of spinal posture during various activities [6]. Clinically, surface scanning is used to evaluate torso shape in patients with deformity [7, 8] or to reverse engineer torso geometry when manufacturing spinal braces [4, 9–12]. However, the uptake of this technology is still to some degree hampered by conflicting results relating to the correlation between surface topography and clinically relevant spinal parameters [7].

In a review of prior studies utilising metrics acquired using surface topography to evaluate spinal deformity indices for scoliosis, Patias *et al* [13] stated that surface metrics cannot be used to describe radiological measurements of spinal deformity in these patients. However, Goldberg *et al* [7] found a strong correlation between spinal deformity angles measured using the Quantec surface measurement system and the coronal Cobb angle measured radiographically for scoliosis patients. These surface parameters were calculated using a line demarcating the spinal column on the topographic image of the patient’s back. Despite this stochastic relationship, Goldberg *et al* [7] found 66% of the variation in visible deformity could be explained by variation in Cobb angle, with the remainder of variation attributed to measurement variability and the dissimilarity between spinal curves and back surface shape. Even so, Goldberg *et al* [7] and Kotwicki *et al* [8] note that the use of surface topography could provide a more comprehensive impression of the trunk deformity than can be obtained radiographically and Goldberg *et al* [7] concluded surface scanning may have potential in patient monitoring as an alternative to radiographic imaging.

The advantages in the clinical use of 3D surface scanning (3DSS) include the availability of hardware that is relatively inexpensive and offers high dimensional accuracy [14], the availability of scanners that are straightforward to operate, and of most importance, the process involves no radiation exposure to the subject being scanned. While Patias *et al* [13] concluded that surface-derived metrics have little correlation with radiographic deformity measurements for paediatric patients, there is much potential for the uptake of surface metrics to characterise changes in spinal posture and alignment of the spinal column without the requirement for repeated clinical imaging throughout the growing years and the subsequent cumulative radiation dose.

The current study was a first step towards gaining efficacy in the use of 3DSS to measure the alignment of the spinal vertebra, the thoracolumbar spinal contours and sagittal alignment, all clinically relevant parameters measured in spinal deformity patients (eg. idiopathic scoliosis, Scheuermann’s kyphosis). The current study sought to establish the efficacy and accuracy of using 3D non-contact surface scanning of the external spinal contours to evaluate spinal curvature and sagittal plane profile of the spinal column.

## Materials and methods

A cohort of adult females were recruited to undergo both magnetic resonance imaging (MRI) and 3D surface scanning (3DSS) in order to correlate surface topography of the spine with the internal position and alignment of the spinal column. To achieve this, the analysis was performed in two phases. Firstly, the position of the spinous processes measured from MRI was compared with the position of the external surface markers demarcating the spinous processes, also measured from MRI–this was carried out to establish whether the position of surface markers could replicate the position of the internal spinal bones. Following this, the 3D positions of the surface markers acquired using 3DSS were compared with the position of these markers measured from the MRI–this was carried out to provide efficacy for the use of 3DSS in characterising spinal contours.

### Subjects

A cohort of 10 healthy adult females, with no prior history of spinal conditions participated in the study after providing written informed consent to participate. The mean age of participants was 25 years (standard deviation, 3.6 years). Participants were asked to wear sportswear, to permit clear visualisation of the posterior torso surface when lying in the lateral decubitus position. To provide an indication of participant body stature, body mass index (BMI) was calculated as body weight (kg) divided by height squared (m2) and compared to healthy BMI range, 18–25 (The Heart Foundation, Australia).

### Imaging

Each participant underwent both 3DSS and MR imaging. An Artec Eva (Artec3D, Luxembourg City, Luxembourg) non-contact scanner was used for the 3DSS. This is a structured light scanner that projects a flashing fringe pattern onto the object of interest. The camera records how this pattern deforms over the object, thereby extracting data on the object size and shape [14]. The 3D position of the point cloud obtained using the scanner is used to reconstruct the surface geometry of the object, in this case the participant’s torso.

MRI scans were performed on a 3-T Philips Achieva clinical MRI scanner. A 3D T2-weighted sequence was utilised (Echo time 1.725 ms, Repetition time 3.82 ms, In-plane resolution 0.708 x 0.708 mm, Slice spacing 0.75 mm) with a field of view from shoulders to femoral heads and a scan duration of approximately 12 minutes. Despite this scan duration, the radiographer reported there was no blurring of the acquired images that could be associated with participant movement (other than respiration).

The participants could not undergo 3DSS while in the MRI scanner because the scanner equipment included metallic parts. Additionally, the MR imaging took place in a busy public hospital radiology department, so it was not possible for participants to be surface scanned in the MRI department and then wheeled into the scanner immediately afterwards. For this reason, the participants first underwent 3DSS while lying on a non-deformable board placed on a hospital plinth in an adjacent room. This substrate was selected to replicate the non-deformable plastic gantry surface on which the participant lay in the MRI scanner.

**Fig 1:**
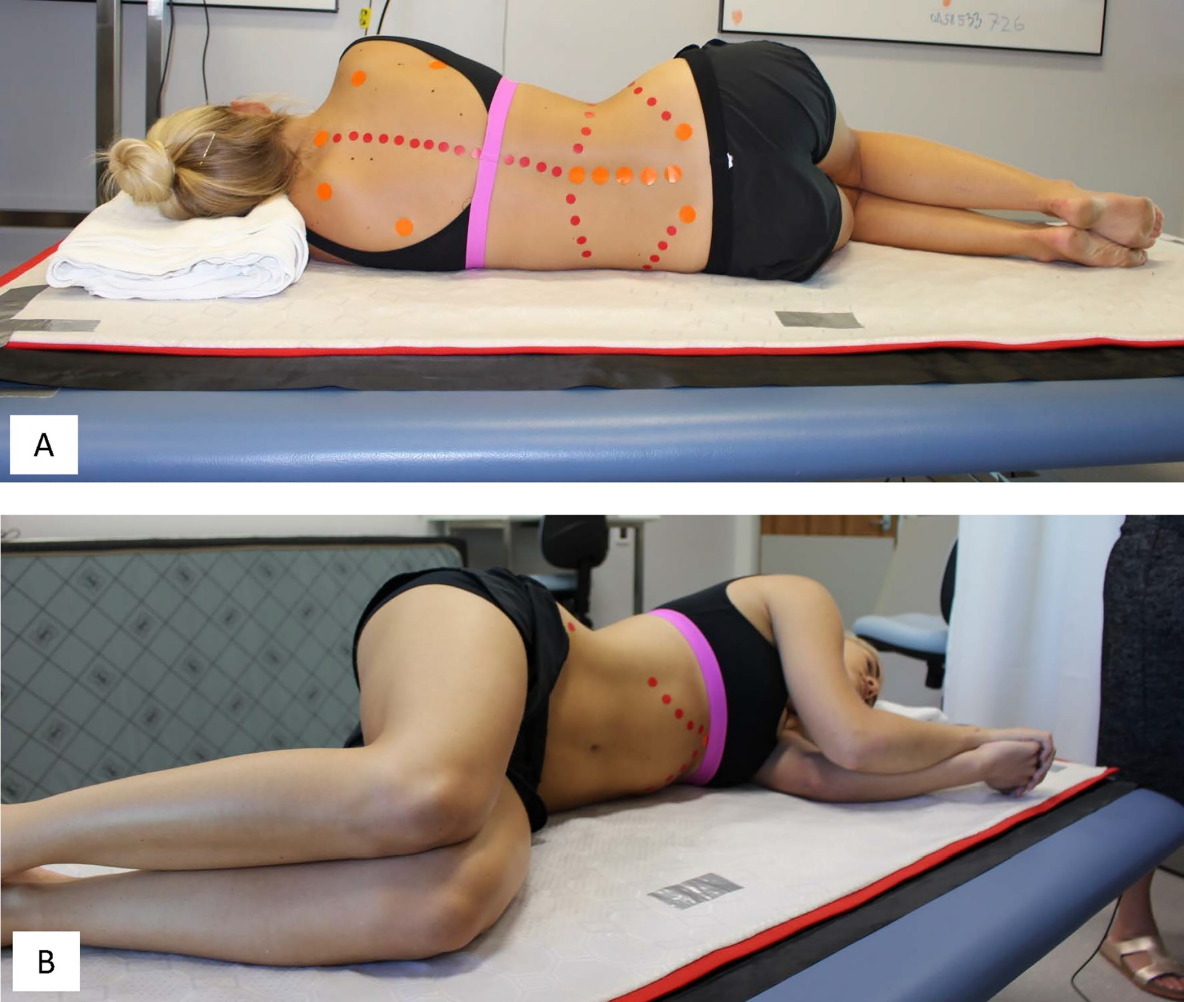
Participant positioned laterally. Participant 10 lying laterally on a mat (white) overlaid on a non-deformable board on the cushioned plinth. A folded hospital blanket provided a regular head support. (A) Participants were asked to position themselves such that their shoulders were ‘stacked’ vertically and hips were similarly aligned vertically. (B) The participant’s femurs and humeri were at a regular angle to the torso (60^o^ and 30^o^, respectively). Consent to publish these photographs was obtained.

Participants were positioned by the study authors to ensure that while lying in the left lateral decubitus position, their shoulders were aligned vertically, their hips were aligned vertically, their legs were aligned such that the femur was at an angle of 60^o^ to the torso, their tibia at 60^o^ to the femur and their feet were in line with the body (Fig 1). (The individual in this manuscript has given written informed consent, as outlined in PLOS consent form, to publish these case details). The participant’s humeri were aligned at an angle of 30^o^ to the torso with hands in front of their face (Fig 1). This positioning ensured the participants had sufficient clearance in relation to the bore diameter of the MRI equipment, to allow the same position to be replicated during MR scanning.

For all participants, a folded blanket provided head support of regular height for both 3DSS and MRI scan positioning (Fig 1). This ensured there was no undesirable muscle strain at the neck and shoulders. The blanket was folded into a similar configuration for each condition to ensure the head was supported at a height of 50–75% of the distance between the shoulder and the lateral margin of the head [15].

**Fig 2:**
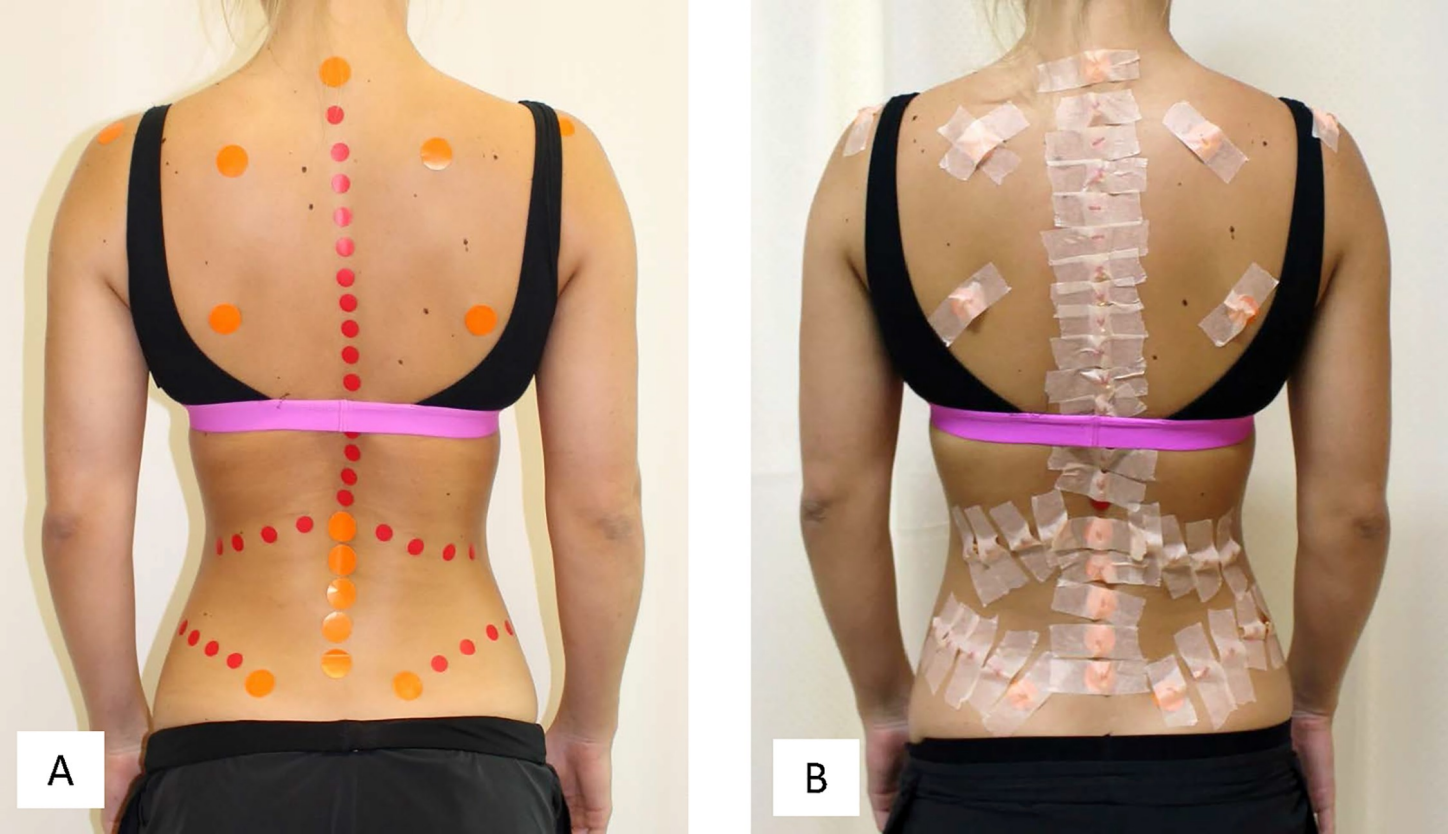
Posterior view of participant. (A) Stickers were attached over bony landmarks on the participant’s spinous processes and back surface to permit spinal anatomy to be clearly demarcated in the 3DSS reconstructions. (B) Fiducial markers were overlaid on the surface stickers and attached with surgical tape to permit registration between landmarks from 3DSS and MRI. Consent to publish these photographs was obtained.

In the first instance, anatomical landmarks on the posterior spinous processes of each participant were demarcated using stickers attached to the skin surface (Fig 2A). Anatomical landmarks were isolated by an experienced physiotherapist (Author, MTI), and stickers were used to highlight anatomy for the thoracic and lumbar spinous processes on the surface scan. Stickers were of a regular size and circular in shape, to permit co-ordinates for the centroid of each sticker to be readily defined when post-processing the 3DSS images.

Once 3DSS was completed, fiducial markers were overlaid on the surface stickers and attached with surgical tape (Fig 2B). The fiducial markers were Vitamin D capsules which have recently been shown to provide excellent intensity contrast for MRI scanning [16]. Fiducial markers highlighted the same anatomical landmarks on the MR images as the surface markers, permitting registration of points and point clouds from both imaging modalities.

The participant was then re-positioned on the MRI gantry, ensuring their shoulder, hip, leg and arm positions resulted in comparable torso alignment to that for the 3DSS.

### Image analysis

**Fig 3:**
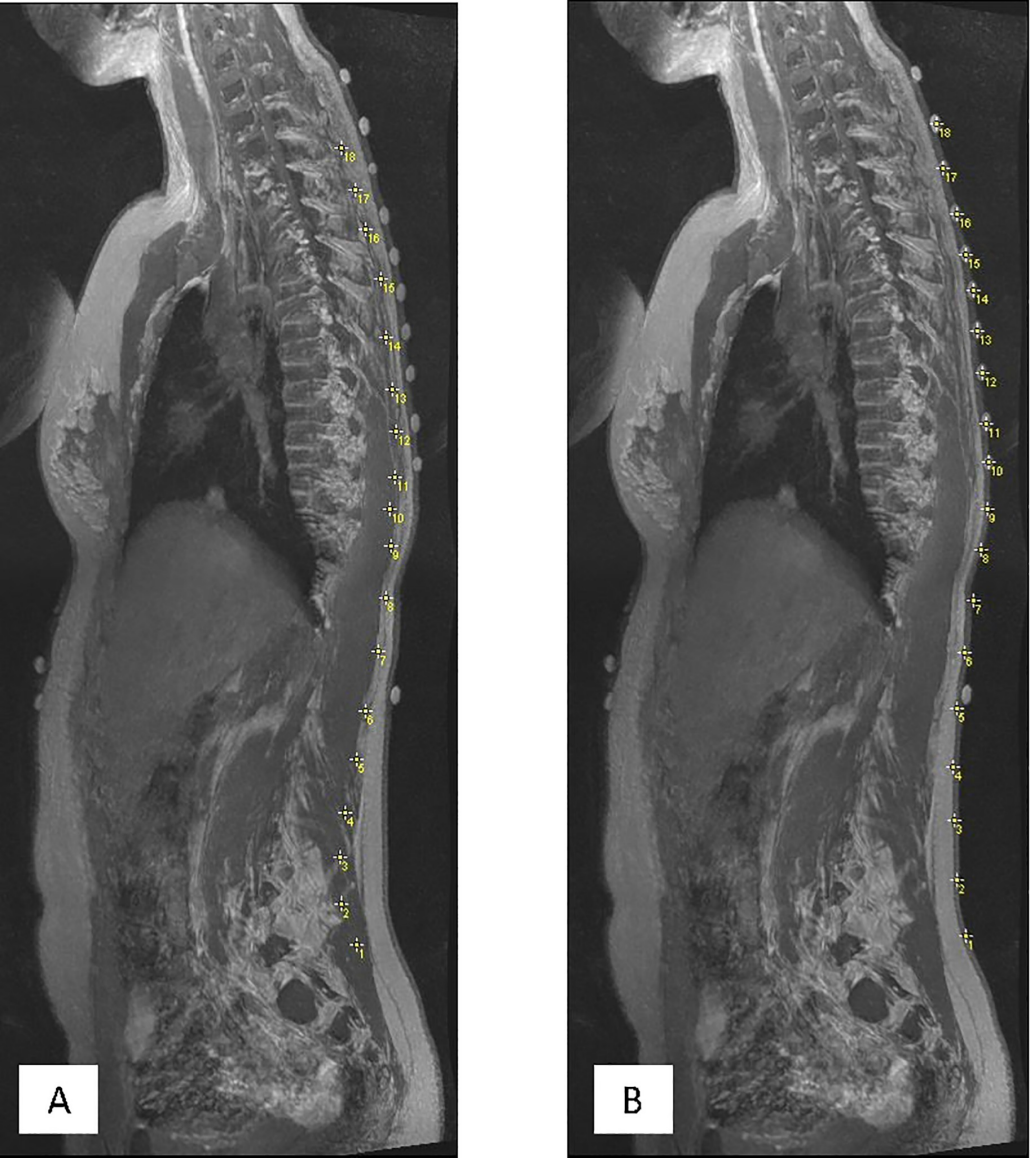
Sagittal plane maximum intensity projection. Sagittal plane projection (ImageJ) of the reformatted MRI data for Participant 9. (A) Yellow crosses demarcate the posterior margin of the spinous processes at each spinal level in the thoracolumbar spine; (B) Yellow crosses demarcate the centre of the fiducial marker attached to the skin surface over the thoracolumbar spine.

In the first phase of analysis, the MRI data for each participant was used to compare the internal and external spinal column curvature. MRI datasets were reformatted to create sagittal reslices (ImageJ, US National Institute of Health open-source software, Maryland, USA; https://imagej.nih.gov/ij/), and using the ImageJ multi-point selection tool, the sagittal view of the dataset was advanced between reslice images to select points on the posterior margin of each spinous process from T1 to L5 (Fig 3A). The position of the fiducial surface markers was similarly selected from the sagittal reslice images to create a second set of co-ordinate points (Fig 3B). Both sets of co-ordinates were exported in text format for analysis.

**Fig 4:**
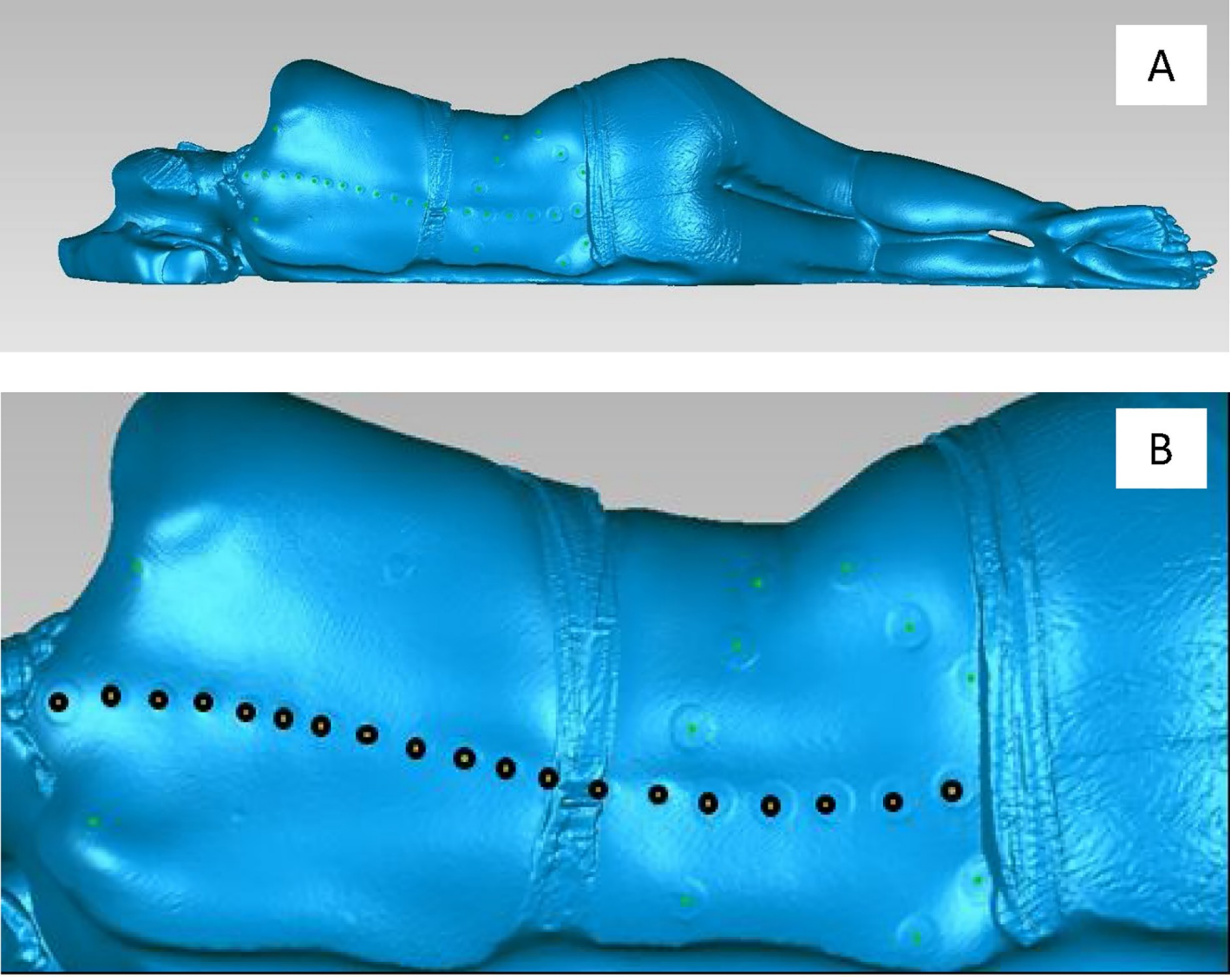
3D surface mesh. 3D surface mesh (STL) of participant 9 showing, (A) Participant viewed from posterior direction, and (B) Point features defined at the centre of the surface markers with the points on the spinous processes highlighted in black.

In the second phase of the analysis, the 3D co-ordinates of the surface markers obtained using 3DSS were compared with the position of the fiducial markers measured from the MRI. To obtain 3D co-ordinates for the surface markers, frames from the raw surface scans were first registered and fused using Artec Studio 10 Professsional (Artec Group, Luxembourg City, Luxembourg), proprietary software used to process point cloud data from the Artec Eva non-contact scanner. The fused triangulated surface mesh was exported in stereolithograhic format (STL) for cleaning and to collect co-ordinate data for the surface markers (Fig 4A) (Geomagic Wrap, 3D Systems, North Carolina, USA, Vsn 2017). The cleaning process involved removal of small components, filling of small holes, and removal of spikes in the surface mesh. While this cleaning process produced an aesthetically favourable surface mesh (Fig 3A), the points of interest on the surface scan were not affected as they included only the surface markers, which had been captured with a smooth surface from the 3DSS. The centroid of the circular surface stickers was then demarcated with a point feature (Fig 4B). These centroids were used to map the external sagittal profile of the spinal column–the co-ordinates were exported as a text file for further analysis.

### Data analysis

Three sets of co-ordinate points were analysed for each participant, representing the sagittal profile of the spinal column using:

- Surface markers from 3DSS
- Surface markers from MRI
- Posterior margin of the bony spinous processes from MRI

A 7^th^ order polynomial was fit to each co-ordinate point profile (Matlab R2017a, Mathworks, Massachusetts, USA) and compared with the raw co-ordinates using a root-mean-squared-error calculation (RMSE). These polynomials were then used in place of the discrete co-ordinate point profiles, to permit analytical comparison of the shape and curvature between the MRI and 3DSS datasets over a regular interval of axially spaced points.

To compare the position of the spinous processes and fiducial markers derived from the MRI datasets, the two profiles were compared qualitatively and quantitatively to determine whether: the two profiles were visually similar; demonstrated a consistent linear distance from one another (indicative of a skin and adipose layer) over the length of the spine; and the curvature (k) of both profiles was similar along the axial length of the spine. Curvature, k, measured the deviation of the profile from a straight line (Matlab R2017a). Curvature was calculated using the points defining each profile, and the analytical curvature calculated using the equation for curvature of plane parametric curves (Eq 1). This was applied using the Matlab function ‘LineCurvature2D.m’ (v1.3.0.0, © 2011, Dirk-Jan Kroon, University of Twente).

$$k=\frac{\left\|y^{\prime }x^{"}-y^{"}x^{\prime }\right\|}{{\left(x^{\prime 2}+y^{\prime 2}\right)}^{3/2}}.$$ Eq 1

To compare the fiducial marker positions derived from MRI with the surface marker positions from 3DSS, the two respective profiles were compared qualitatively to assess their visual similarity and whether the thoracic and lumbar sagittal kyphosis and lordosis, respectively, were comparable between the scanning modalities. The RMSE between the two profiles was calculated to assess the mean linear distance between the profiles along the axial length of the spine. Curvature, k, was calculated for both profiles and compared over the length of the spine. A two sample Kolmogorov-Smirnov test was used to assess whether the MRI and 3DSS surface marker profiles were from the same distribution. 22 MRI and 3DSS co-ordinate points at regularly spaced (10mm apart) locations along each profile were compared and significance calculated using IBM SPSS Statistics (Version 23), with a significance level 0.05. The null hypothesis was H_0_: both samples are from populations with the same distribution.

## Results

The BMI for Participants 3–10 fell within the healthy range, 18–25, while Participant One’s BMI was 16, and classified as ‘Underweight’ and Participant Two’s BMI was 28, and classified as ‘Overweight’.

The RMSE for the 7^th^ order polynomial fit to the MRI-derived spinous process profile (error between points and polynomial) ranged from 0.51mm to 0.96mm (Median RMSE 0.90mm) and for the MRI-derived fiducial marker profile ranged from 0.38mm to 1.15mm (Median RMSE 0.725). Regarding the 3DSS-derived surface marker profile, the RMSE for the 7^th^ order polynomial ranged from 0.17mm to 1.02mm (Median RMSE 0.30mm).

### Comparing surface marker position with spinous process position: MRI measurements

**Fig 5:**
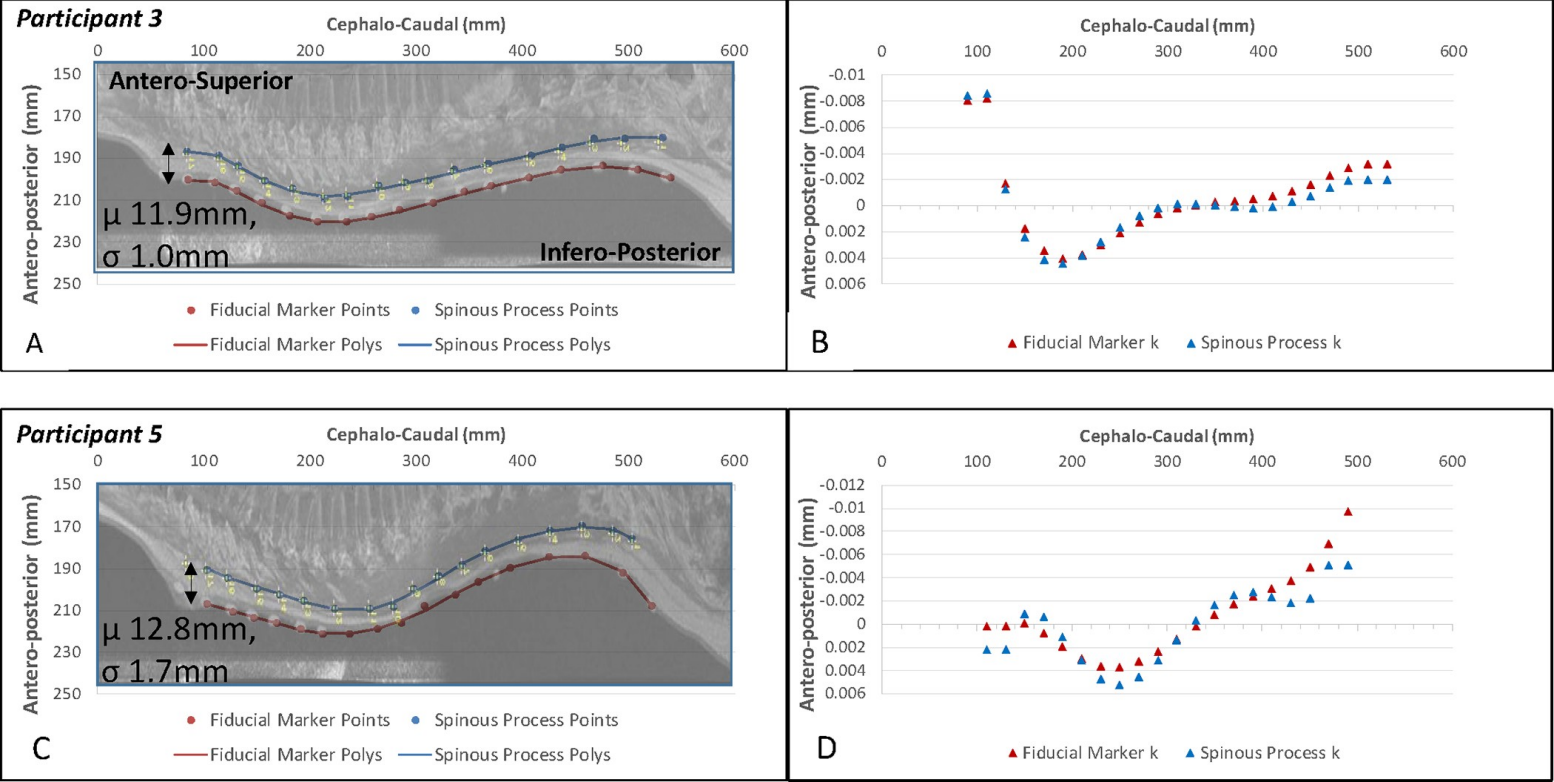
Position of spinous processes and surface fiducial markers. Position of spinous processes (Blue) and surface fiducial markers (Red), viewed in the sagittal plane, showing a comparison for, (A,B) Participant 3 and (C,D) Participant 5 as an example of the similarity in sagittal curvature and the consistent offset between the profiles (A,C). The curvature, k, over the length of the spine is shown in B and D. The reformatted sagittal plane image of the participant’s spine is overlaid as a reference. (Note, because the x-y co-ordinate axes on these charts are not anatomically scaled, these MRI images are ‘stretched’ to overlay on the charted points.) μ = mean distance between profiles, mm; σ = standard deviation for the mean distance, mm.

In comparing the position of the spinous processes and fiducial markers measured from the MRI datasets, the position of the surface fiducial markers showed a similar profile (Fig 5A and 5C) and curvature (Fig 5B and 5D) to that of the spinous processes. The results for all 10 participants are shown in S1 Fig and for two representative participants in Fig 5. In viewing charts for all participants, it was apparent that, with the exception of Participant two, the variation in contour along the thoracic and upper lumbar spine was similar (Fig 5A and 5B). The exception was at the lower lumbar spine, particularly at L5 and in some cases at L4, where the surface fiducial marker profile was not as consistently offset from the spinous processes, resulting in a larger distance between the profiles. This was a result of the thicker region of soft tissue, in terms of both muscle and adipose tissue, around the upper gluteal muscles.

The position of the two profiles for the most part demonstrated a consistent linear distance over the length of the spine (S1 Fig, Fig 5), with a mean difference between the spinous process and fiducial marker profiles of 13.5mm over all participants. This mean difference represented the cumulative contribution of the mean thickness of skin/adipose/muscle/ligament tissue between the spinous processes and the outer skin surface as well as the 4mm distance from skin surface to oil capsule centre. Of more importance, for individual participants, the standard deviation (σ) of this mean ranged from 1.0–4.0mm for nine of the 10 participants, with Participant Two the exception having a σ = 7mm (S1 Fig).

Regarding Participant Two, her body shape and composition was such that she fell at the upper limit of the BMI range included in the study. Consequently, the region between the spinous processes and skin surface included a comparatively larger region of adipose tissue (visible as high intensity pixels on T2-weighted MRI) than the remaining participants. This adipose layer and the associated skin movement artefact resulted in a dissimilar sagittal profile for the spinous processes and MRI-visible fiducial markers.

### Comparing surface marker position: MRI vs 3DSS

**Fig 6:**
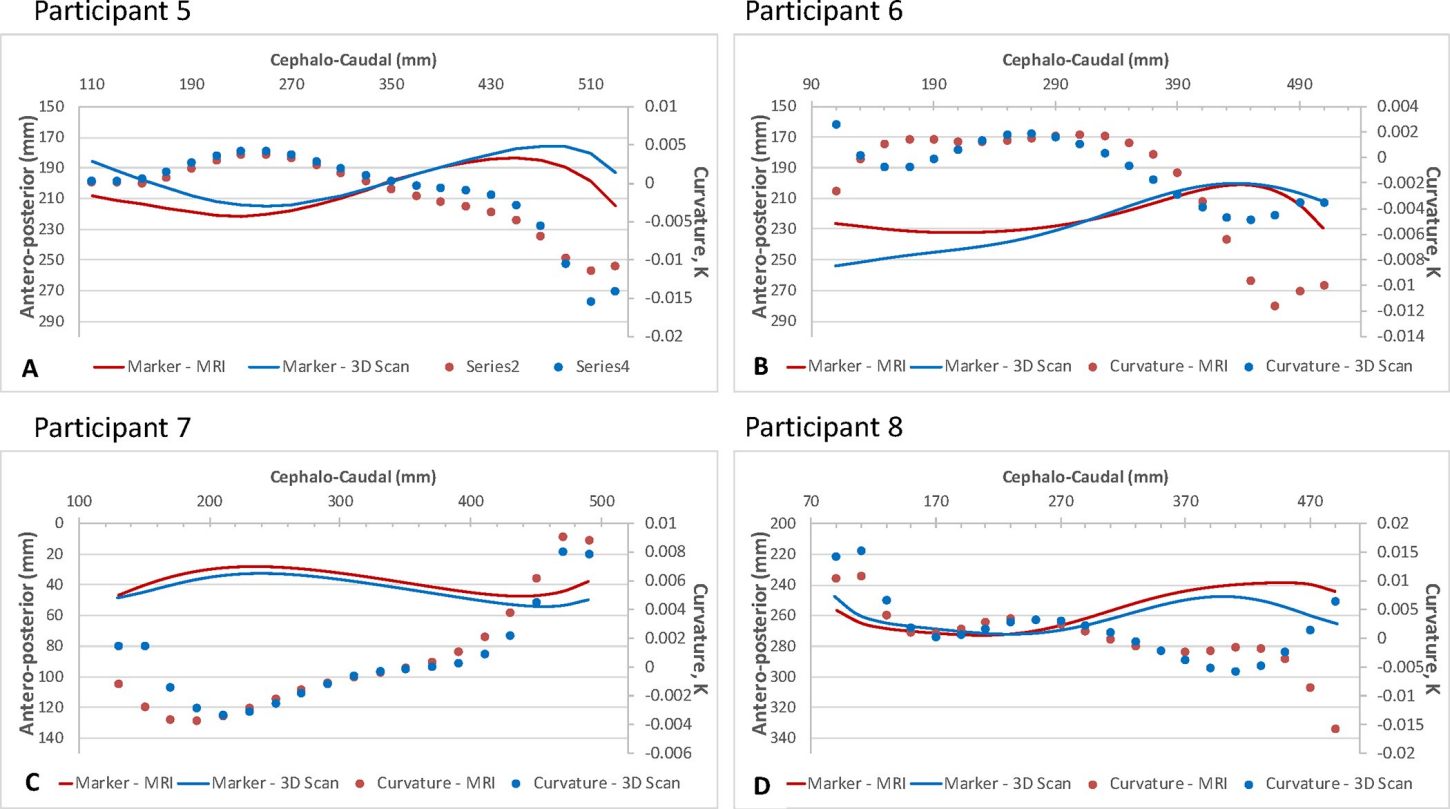
Comparison of the MRI and 3DSS-derived surface marker positions and spinal curvature. Comparison of the MRI- (Red) and 3DSS-derived (Blue) surface marker positions and spinal curvature viewed in the sagittal plane (x axis = axial direction along spin, y axis = lateral direction along spine). Solid lines show the position of the markers in the antero-posterior/caudo-cephalic directions (mm); Filled circles show the variation in curvature, k, along the caudo-cephalic spine. Participant five (A) and seven (C) demonstrated a similar profile and curvature, k, between 3DSS and MRI. Participants six (B) and eight (D) demonstrated differing profiles. Note: The co-ordinates of the sagittal profiles (ie. x and y values) were based the co-ordinate system for the MRI dataset–the 3DSS profiles were transposed to this co-ordinate system.

Comparing the MRI-derived fiducial marker profile with the 3DSS-derived surface marker position (Fig 6), the RMSE between the positions of the surface markers had a mean value of 9.8mm (σ = 4.2mm) when compared over the entire thoracolumbar spine. However, these profiles tended to show better agreement over the lumbar spine with a mean RMSE of 6.0mm (σ = 4.7mm) compared to the thoracic spine, with a mean RMSE of 10.9mm (σ = 5.4mm).

Examples of participant profiles that showed similar and dissimilar curvature are shown in Fig 6. S2 Fig shows a comparison between the MRI- and 3DSS-derived profiles for all 10 participants. For seven of the participants, the change in curvature (k) over the thoracolumbar spine was similar (S1 Fig), with the MRI- and 3DSS-derived profiles demonstrating a similar k. Kolmogorov-Smirnov test results for two independent samples showed no significant difference between populations when comparing all MRI- and 3DSS-derived marker positions for all 10 patients (p = 0.215). However, when comparing the 22 profile points for individual participants, three participants demonstrated dissimilar profiles (p<0.05, S2 Fig).

For participant five (Fig 6A), k was similar over the cephalo-caudal length of the spine. As was seen when comparing the position of the spinous processes with the surface fiducial markers, the added soft tissue thickness in the upper gluteal region produced some variation in k in the lower lumbar spine, near L4 and L5. Similarly, for participant seven (Fig 6C), the MRI- and 3DSS-derived profiles were visibly similar across the length of the spine, with some variation in k observed at the cephalic and caudal limits, which was in part due to discontinuities in the curvature algorithm at the limits of the polynomial fit and also due to the gluteal soft tissues. For both these participants, the similarity in MRI- and 3DSS-derived profiles was such that the sagittal contours of the spine, in terms of kyphotic and lordotic spinal curves, were clearly identifiable from the 3DSS results.

Considering participant six (Fig 6B), the MRI- and 3DSS-derived profiles were dissimilar, particularly in the upper thoracic and lower lumbar spine. Similarly, the curvature across the cephalo-caudal length of the spine differed between the profiles. While the MRI- and 3DSS-derived contours for participant eight were dissimilar in the lower lumbar region (Fig 6D), the curvature across the thoracic spine (Fig 6D, cephalo-caudal dimension <370mm) showed a good match between profiles. In comparing the curvature over the lumbar spine, however, both the value and direction of curvature differed between the profiles for this participant.

**Fig 7:**
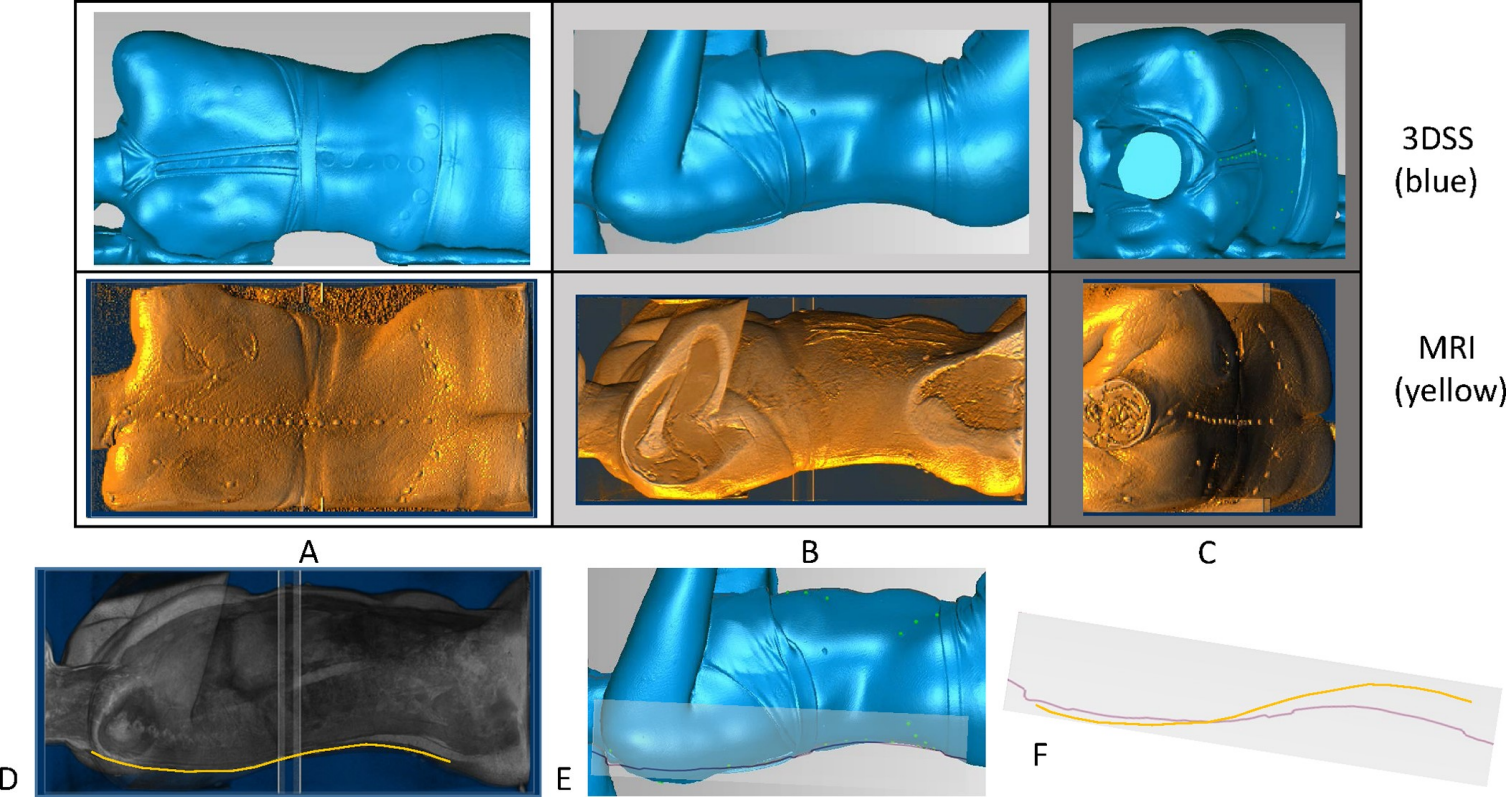
Posterior, Right lateral and Superior view of the skin envelope over the torso. Posterior (A), Right lateral (B) and Superior (C) view of the skin envelope over the torso for participant 8, reconstructed from 3DSS (blue) and MRI (yellow) data. The difference in participant position, body shape and shoulder/hip orientation is evident in all three orientations, and highlighted by comparing the trajectory of the fiducial surface markers on the spinal column in both A and C. Comparing a line demarcating the sagittal profile of surface markers on the MRI (D, yellow) and 3DSS (E, purple) it is evident that these profiles differ both in shape and curvature (F).

Regarding the three participants demonstrating dissimilar MRI and 3DSS profiles, a comparison of the shape and position of their torso during both scanning processes highlighted that the participant position was not consistent between the MRI scan and the 3D scan. Fig 7 shows a comparison of the body shape, position and shoulder/hip orientation for Participant 8 (Fig 7A–7C), highlighting how these variations between MRI (Fig 7D) and 3DSS (Fig 7E) can result in a differing sagittal profile (Fig 7F). Conversely, for the remaining seven participants, a comparison of this surface shape demonstrated a consistent position, with shoulders and hips aligned perpendicular to the table and the shoulder and pelvic orientation comparable.

## Discussion

This study first sought to determine whether fiducial surface markers placed over the spinous processes could map the internal position of the spinous processes in the thoracolumbar spine. Once established, the study results demonstrated the efficacy of using 3D surface scanning to reproduce and map the position of these surface markers and to provide a surrogate for clinical imaging in describing the sagittal plane profile of the spinal column.

While the current study included a relatively small number of participants, the inclusion criteria were defined to recruit participants with similar body stature and demographics and study results demonstrated consistent patterns for these participants. Furthermore, prior studies of spinal contour have incorporated similar participant numbers with consistent results [17, 18]. Prior studies have suggested that the use of 3DSS is not reliable in mapping clinically relevant coronal plane spinal contours [7, 8, 19], therefore, the focus of the current study was a comparison of sagittal plane profile. Sagittal spinal profiles are relevant in assessing spinal balance, both for paediatric and adult patients, and are typically assessed using plain-radiographs. As such, the assessment of a surrogate imaging technique which does not involve exposing patients to ionising radiation was appealing. While plain radiographs or computed tomography scans have historically been the imaging modality of choice when investigating bony anatomy, MRI is increasingly being used in modern patient cohorts as it does not expose the subject to ionising radiation [20–22], yet still provides reliable anatomical measurements.

In addressing the first of the above study questions, regarding whether fiducial surface markers could map the spinous process position in the sagittal plane, for the majority of participants (nine of 10) the results showed a consistent distance between the MRI-derived spinous process and surface marker profiles from T1 down to the mid-lumbar spine. This consistency was evident both qualitatively, in visually comparing the profiles, and quantitatively, with the quantitative mean distance between the profiles demonstrating a variation in this distance (1-4mm) that was an order of magnitude smaller. Ghaneei *et al* [23] recently demonstrated that fatty tissue may mask the location of underlying bony anatomy when using surface scanning to assess spinal bone position, a trend that was similarly observed in the current study with less consistency observed between the spinous process and surface markers in the lower lumbar spine, over the gluteal muscles, and for participant 2 (BMI = 28). Findings in the current study were in keeping with the findings of Morl and Blickham [17], who used MRI to assess the alignment between fiducial markers overlaid on the L3 and L4 spinous processes for nine healthy males. With increasing degrees of sagittal plane rotation they found a strong positive relationship between the position of both marker and spinous process. Similarly, in comparing sagittal plane contours over the thoracolumbar spine for 10 adolescent spinal patients, Schmid *et al*. [18] observed comparable profiles for reflective markers placed over the spinous processes (measured using motion analysis system) compared to the anatomical location of the spinous processes measured from biplanar radiography.

Results for the range of BMI included in this study suggest that for persons with a higher BMI and a greater thickness of adipose tissue separating bony anatomy from the visible skin surface, extracting information on the position of the internal spinal bones using visible surface anatomy becomes less reliable. It is difficult to palpate bony spinal anatomy on persons of such stature and therefore reasonable that it is similarly difficult to demarcate surface anatomy using 3D surface reconstructions. Of note is that the participants in both the prior studies demonstrating agreement between surface and internal spinal anatomy [17, 18] were all of a healthy to underweight BMI.

For seven of the participants in this study, the sagittal profile of the MRI- and 3DSS-derived fiducial marker positions demonstrated similar spinal contours, in terms of thoracic kyphosis and lumbar lordosis, as well as similar variation in curvature over the thoracolumbar spine. This was also apparent when comparing the 3D reconstructions of the skin envelope from both MRI and 3DSS. Results for the three participants for which the profiles did not match highlighted the importance of participant positioning. Due to logistical constraints associated with the hospital Radiology service, participants were re-positioned laterally on the MRI gantry after undergoing 3DSS in an adjacent room. While the MR Radiographer and participant were instructed to ensure the same position was achieved for both imaging modalities, there was by necessity some error in this process.

## Conclusion

The 3DSS technique employed in this study is capable of capturing the location of specific features on the skin surface with a high level of accuracy. Comparison of the observed trends for change in vertebral positioning measured from MRI and 3DSS, suggested the surface markers may provide a useful method for measuring internal changes in sagittal curvature or skeletal changes. Application of this may be to monitor spinal changes that occur due to growth. This method however is limited to application in persons for whom bony anatomy of interest can be palpated, thus making it possible to apply and 3DSS surface markers reliably.

## Supporting information

S1 Fig **Position of spinous processes (Blue) and surface fiducial markers (Red) for participants 1 through 10, viewed in the sagittal plane.** The unbroken lines indicate the 7^th^ order polynomial fit to the marker points. (Note: for participant 7, the Anterior-posterior co-ordinate system was flipped.)
(PPTX)

S2 Fig **Comparison of the MRI- (Red) and 3DSS-derived (Blue) surface marker positions and spinal curvature for participants 1 through 10, viewed in the sagittal plane.** Unbroken lines show the position of the markers in the antero-posterior/caudo-cephalic directions (mm); Filled circles show the variation in curvature, k, along the caudo-cephalic spine. Participants two and six are highlighted with Red, indicating the sagittal profile was dissimilar over the thoracolumbar spine. Participant eight is highlighted in Yellow, indicating the sagittal profile was dissimilar in the lumbar spine.
(PPTX)
