# Supplementary material for: Predicting spinal profile using 3D non-contact surface scanning: Changes in surface topography as a predictor of internal spinal alignment
Source: PLoS One. 2019 Sep 26;14(9):e0222453. doi: 10.1371/journal.pone.0222453 (PMC6762190; doi:10.1371/journal.pone.0222453)
Supplement: S1 Fig — Position of spinous processes (Blue) and surface fiducial markers (Red) for participants 1 through 10, viewed in the sagittal plane. The unbroken lines indicate the 7th order polynomial fit to the marker points. (Note: for participant 7, the Anterior-posterior co-ordinate system was flipped.) (PPTX) [file pone.0222453.s001.pptx]

## Slide 1
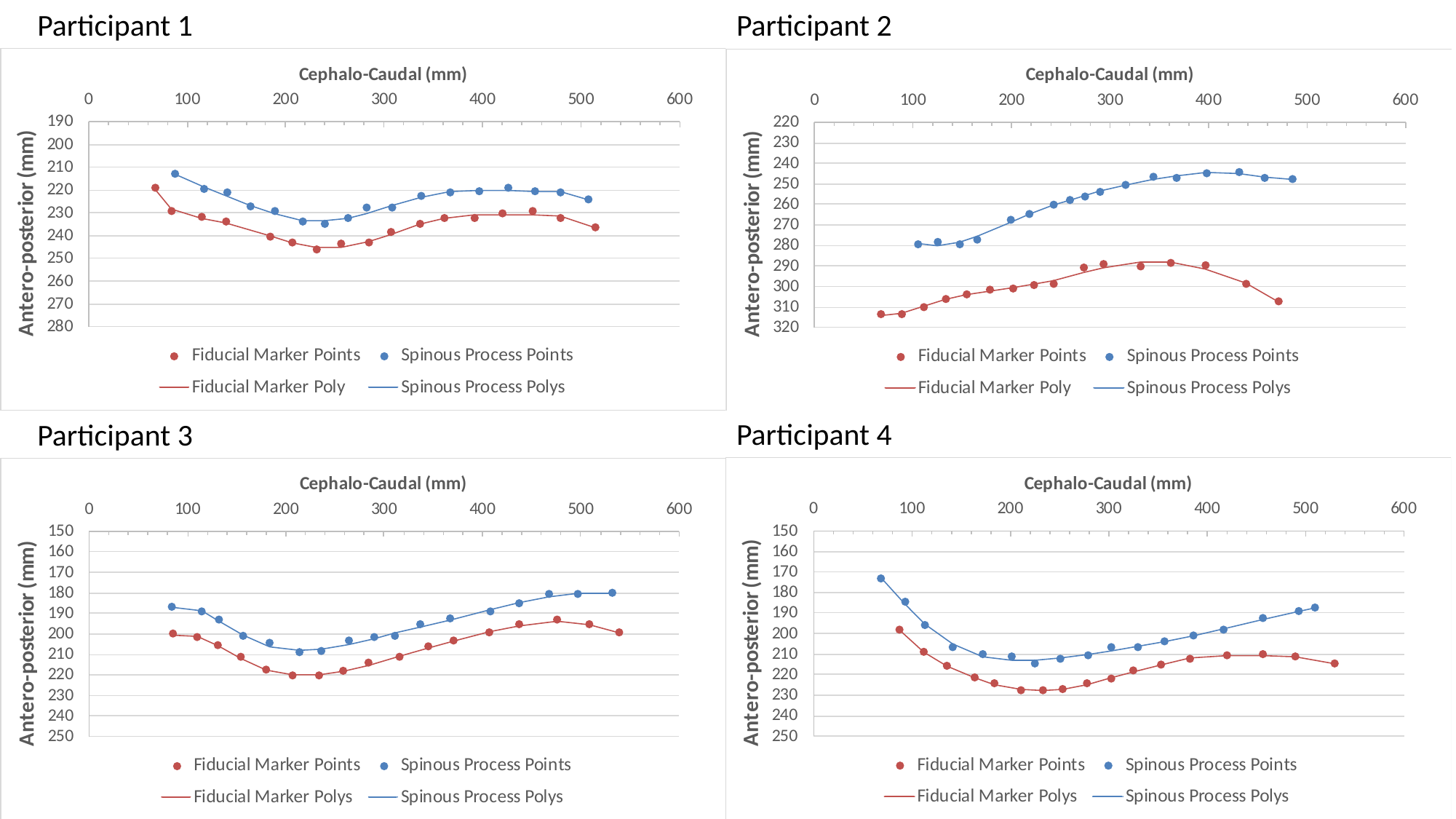

Participant 1
Participant 2
Participant 4
Participant 3

## Slide 2
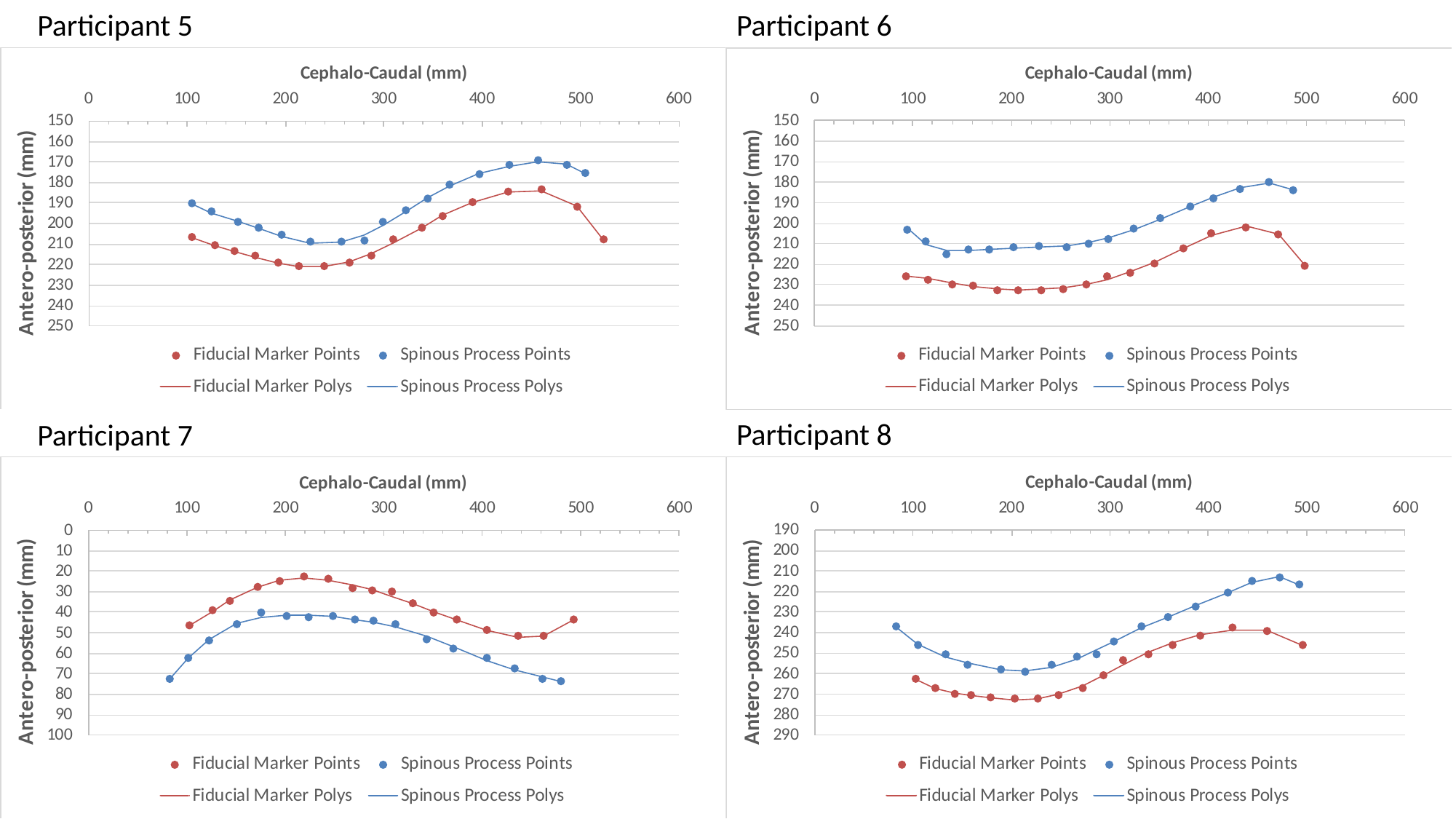

Participant 5
Participant 6
Participant 8
Participant 7

## Slide 3
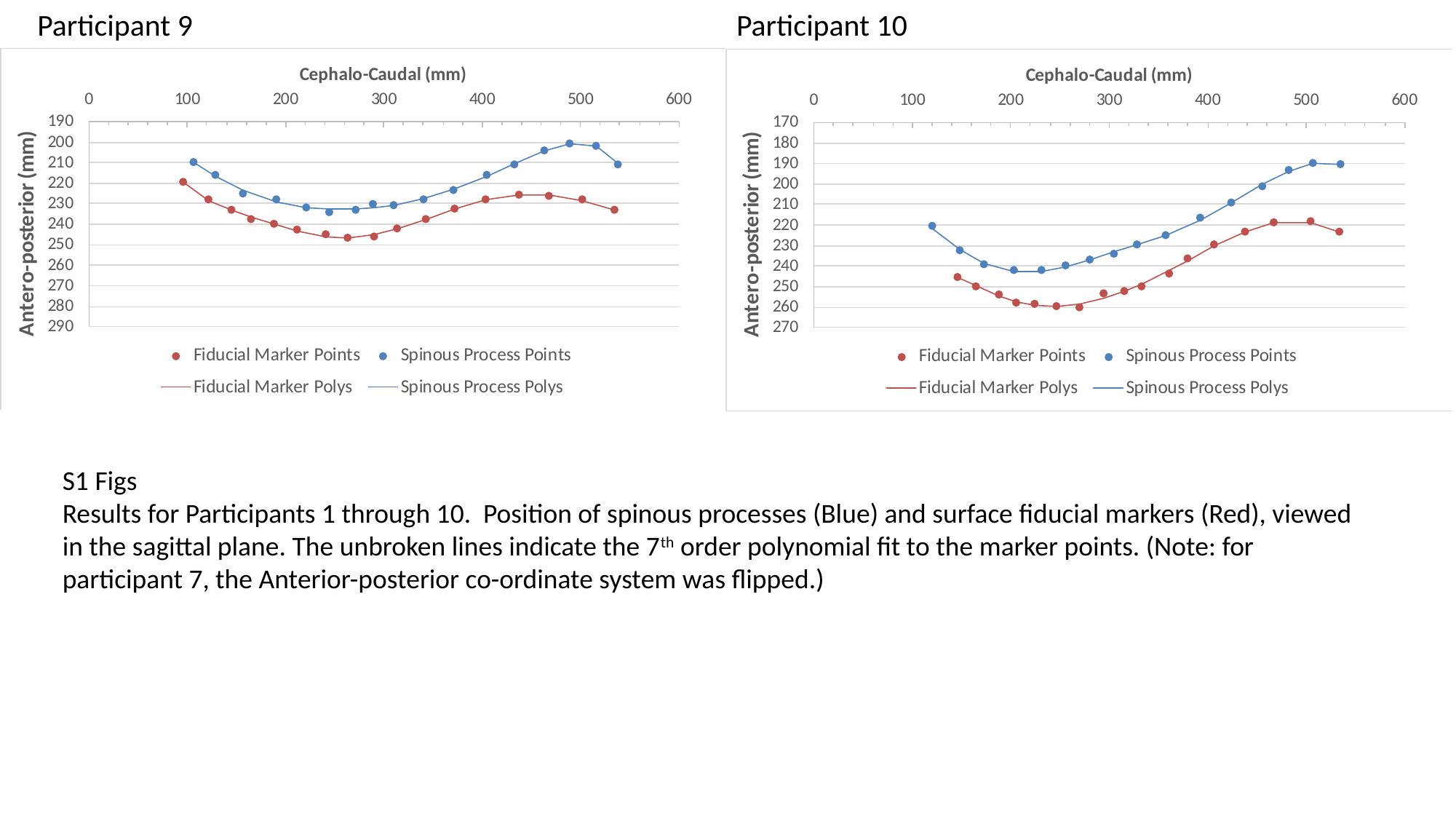

Participant 9
Participant 10
S1 Figs
Results for Participants 1 through 10. Position of spinous processes (Blue) and surface fiducial markers (Red), viewed in the sagittal plane. The unbroken lines indicate the 7th order polynomial fit to the marker points. (Note: for participant 7, the Anterior-posterior co-ordinate system was flipped.)
