# Supplementary material for: Predicting spinal profile using 3D non-contact surface scanning: Changes in surface topography as a predictor of internal spinal alignment
Source: PLoS One. 2019 Sep 26;14(9):e0222453. doi: 10.1371/journal.pone.0222453 (PMC6762190; doi:10.1371/journal.pone.0222453)
Supplement: S2 Fig — Comparison of the MRI- (Red) and 3DSS-derived (Blue) surface marker positions and spinal curvature for participants 1 through 10, viewed in the sagittal plane. Unbroken lines show the position of the markers in the antero-posterior/caudo-cephalic directions (mm); Filled circles show the variation in curvature, k, along the caudo-cephalic spine. Participants two and six are highlighted with Red, indicating the sagittal profile was dissimilar over the thoracolumbar spine. Participant eight is highlighted in Yellow, indicating the sagittal profile was dissimilar in the lumbar spine. (PPTX) [file pone.0222453.s002.pptx]

## Slide 1
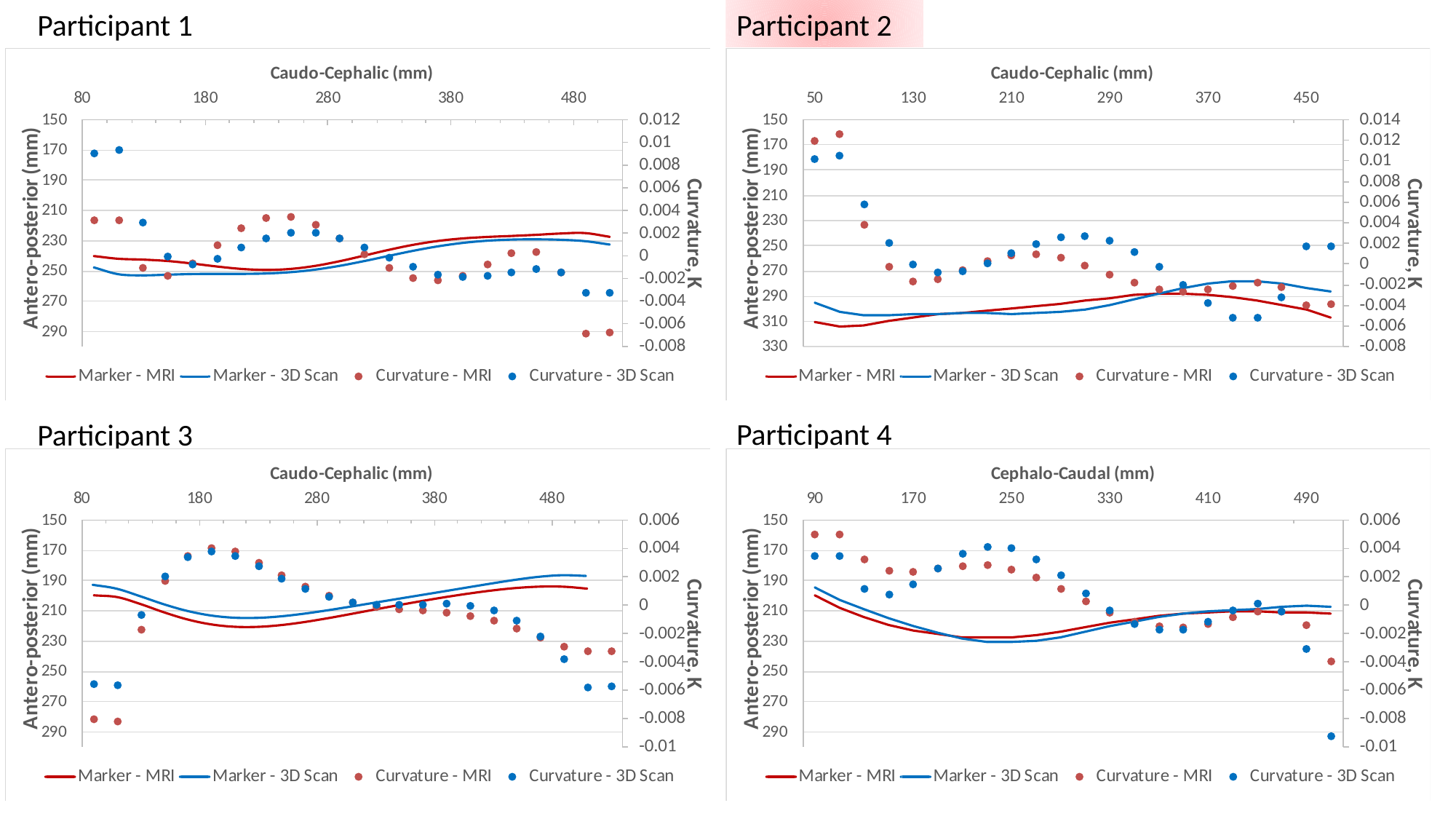

Participant 1
Participant 2
Participant 4
Participant 3

## Slide 2
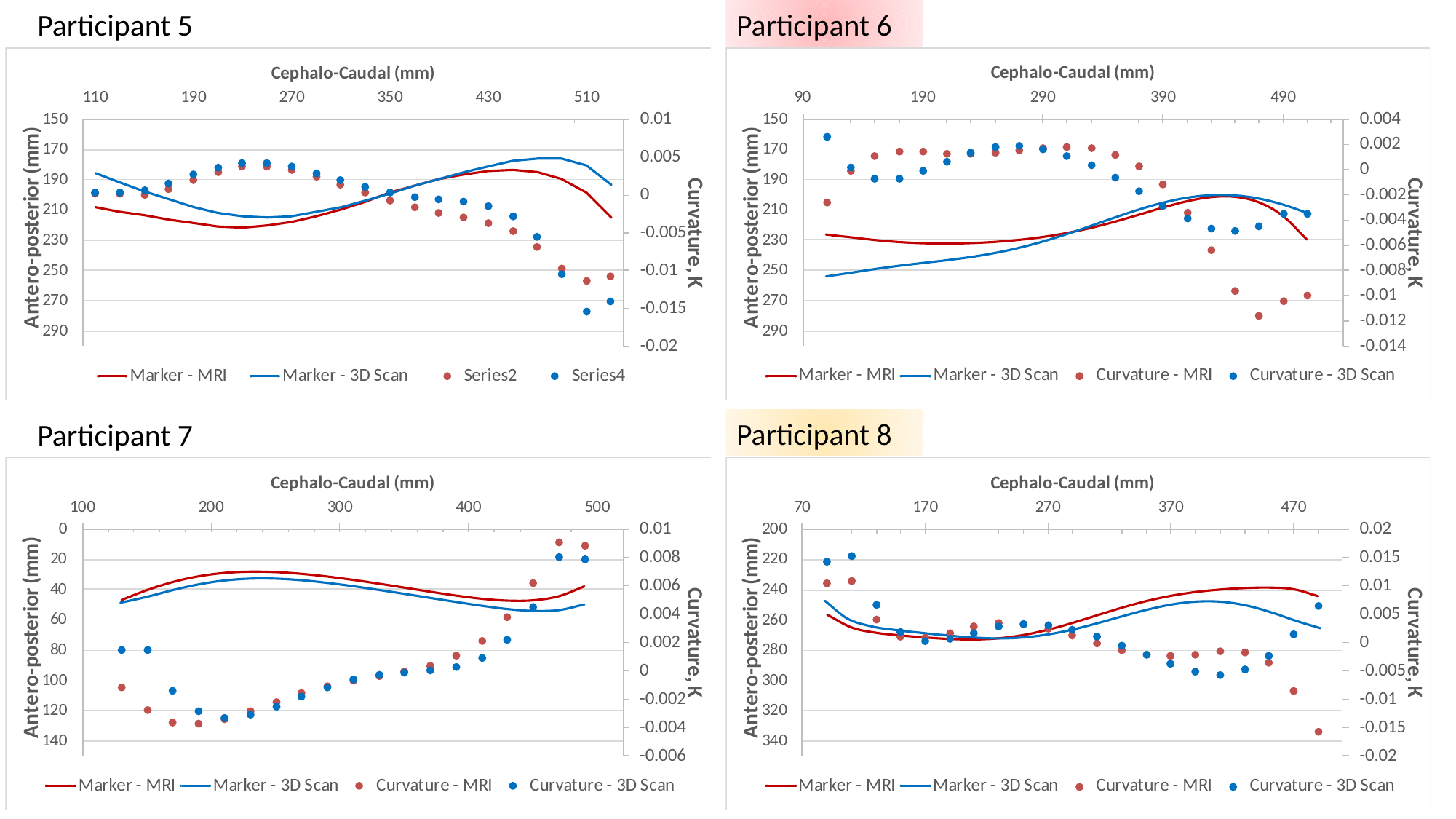

Participant 5
Participant 6
Participant 8
Participant 7

## Slide 3
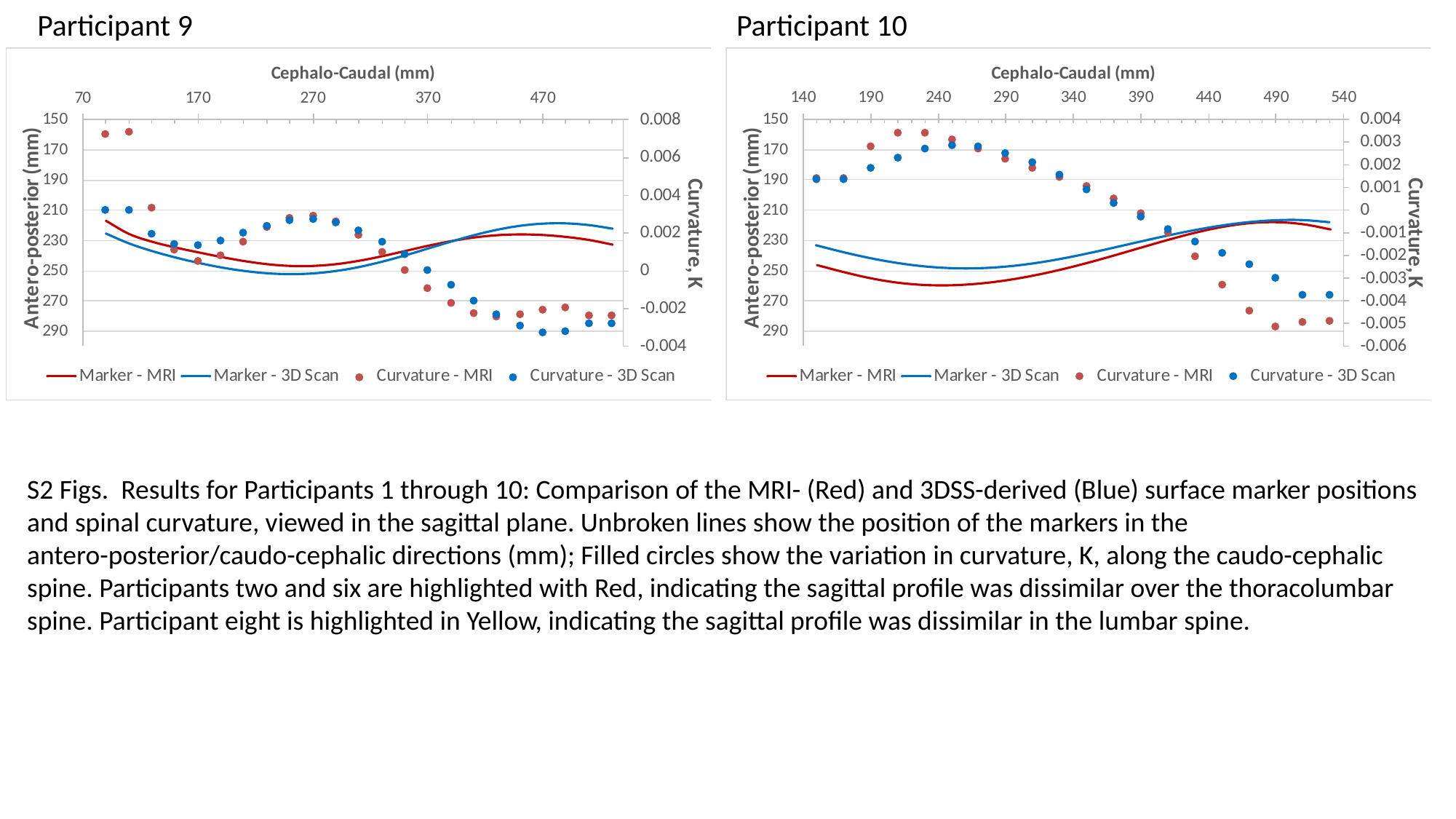

Participant 9
Participant 10
S2 Figs. Results for Participants 1 through 10: Comparison of the MRI- (Red) and 3DSS-derived (Blue) surface marker positions and spinal curvature, viewed in the sagittal plane. Unbroken lines show the position of the markers in the antero-posterior/caudo-cephalic directions (mm); Filled circles show the variation in curvature, K, along the caudo-cephalic spine. Participants two and six are highlighted with Red, indicating the sagittal profile was dissimilar over the thoracolumbar spine. Participant eight is highlighted in Yellow, indicating the sagittal profile was dissimilar in the lumbar spine.
